# The Serum Treatment of Cerebro-Spinal Meningitis

**Published:** 1908-12-19

**Authors:** 


### DISEASES OF CHILDREN.

### THE SERUM TREATMENT OF CEREBRO-SPINAL MENINGITIS.

The discussion on cerebro-spinal meningitis at the last meeting of the British Medical Association, clearly shows that the prospect of curing this disease by the intradural injection of a specific serum is decidedly hopeful, and that it affords almost the only hope of obtaining good results in this most dangerous and fatal affection.

The mortality of the epidemic type of cerebro-spinal meningitis varies in different epidemics from 50 to 75 per cent., but may reach the high percentage of 95 at the beginning of an outbreak. Even in the post-basic meningitis of infancy the results are very bad under two years of age (50 to 75 per cent. fatal). Although there is very strong evidence that the infantile variety is due to the same organism as the epidemic disease, there are frequently found minor differences, for instance in agglutination reactions. It may possibly be found necessary in the future to produce a serum from the special strain, in order to get the best results. At present we may regard the cases as of the same causation, and treat them in the same way.

There are several varieties of serum on the market, prepared by Flexner and Jobling, Kolle and Wassermann, Ruppel, Jochmann, and Burroughs and Wellcome. The first two are those most commonly used and apparently the most reliable. The serum of Flexner and Jobling is prepared from horses immunised by injections derived from many strains of meningococci. It takes from four to five months to prepare a serum of sufficient potency. Kolle and Wassermann prepare their serum by immunising horses and goats with killed cultures first, and finally with living cultures of various selected strains of meningococci. The strength of the serum is estimated according to its agglutinative and opsonic, or bacteriotropic, powers. Both these sera are anti-bacterial or bacteriolytic, and probably possess some antitoxic powers, though Kolle does not claim this for his preparation.

Under strict antiseptic precautions lumbar puncture is done in a suspected case. Chloroform may be given if necessary. If the fluid obtained is cloudy or purulent, proceed with the serum treatment without waiting for the results of microscopic and cultural examination of the fluid, for it is of importance that time should not be lost, and so far the intradural injection has proved harmless in cases which have subsequently been shown not due to the meningococcus. It is advisable to use a Quincke's needle, and to withdraw all fluid which comes away readily after raising the body to the semi-erect posture with the head flexed. If the fluid is so thick and purulent that it will not flow, inject frequent syringefuls of normal saline fluid, and allow it to flow out again, thus washing out the lower part at any rate of the vertebral canal. The serum must be warmed before injection and allowed to flow in slowly, during several minutes, assisted by elevation of the hips and lowering the head. It is not necessary to remove much fluid before injecting, and more may be injected than is removed. Even a "dry" puncture does not contra-indicate injection, provided that the needle is undoubtedly in the canal.

The dose for an adult is 30 c.c. It may be repeated in a severe case in twelve hours, and daily until the temperature falls, and the nervous symptoms abate. A single injection may prove sufficient. Big doses are much more efficacious than repeated small ones, and are apparently harmless.

The effects on the cerebro-spinal fluid are that the meningococci are reduced in number and vitality to such an extent that they stain with difficulty, and the polymorphonuclear cells are reduced in number both in this fluid and in the blood. The effects on the patient in a satisfactory case are that the temperature falls four or five degrees within twenty-four hours, and may not rise again. The nervous symptoms rapidly improve, consciousness returns, prostration is less, and pulse and breathing are better. The rigidity of the neck and stiffness of the extremities are the last signs to disappear. Skin rashes may develop as after diphtheria antitoxin.

The vitality of the cocci is diminished, and their capacity for phagocytosis is increased. Thus, the inflammation is arrested. The serum must be fairly strong and brought into direct contact with the cocci. It is consequently of no value when injected subcutaneously, and it is of importance to remove fluid from the canal before injection.

Statistical results afford definite proof of the value of Flexner's serum. Flexner found the mortality of 442 cases reduced to 33.3 per cent.; or, if forty-nine cases were excluded which died within twenty-four hours, before the serum had time to act, only 25 per cent. Another striking feature is the better results from early injections. If used before the fourth day the mortality is under 15 per cent., 22 per cent. if given on the fourth to the seventh day; and 36 per cent. if later. The fever subsided by crisis in one-fourth, by lysis in the rest. The duration was shortened to an average of eleven days. Relapses occurred in about 5 per cent.; few were fatal. Ker found the mortality of 30 cases similarly treated was 43 per cent., as against 80.5 per cent. of 108 non-serum cases. Robb's results were a mortality of 30 per cent. of 90 cases, as against 72.3 per cent. of non-serum cases; and a total disappearance of the chronic, emaciating, slowly progressive cases. McKenzie and Martin, of Glasgow, obtained six recoveries out of sixteen cases treated by intradural injections of human serum. In two the serum was prepared from the patient's own blood, and in fourteen from the blood of recovered patients.

These results are sufficiently satisfactory to warrant the careful systematic employment of this method of treatment in all cases of meningococcal meningitis, until its value can be accurately ascertained. Even under two years of age Emmet Holt points out that Flexner's results were nineteen deaths out of forty-one cases, a mortality of 46.3 per cent., whereas he himself had had a mortality of 90 per cent. in sixty-one cases under this age.

